# Democratizing cost-effective, agentic artificial intelligence to multilingual medical summarization through knowledge distillation

**DOI:** 10.1038/s41598-025-10451-x

**Published:** 2025-07-29

**Authors:** Chanseo Lee, Sonu Kumar, Kimon A. Vogt, Muhammad Munshi, Panindhra Tallapudi, Antonia Vogt, Hamzeh Awad, Wasim Khan

**Affiliations:** 1 Sporo Health, Boston, MA USA; 2 Department of Anesthesiology, Yale School of Medicine, New Haven, CT 06520 USA; 3 Girton College, University of Cambridge, Cambridge, UK; 4 Faculty of Allied Medical Sciences, Middle East University, Amman, Jordan; 5 Department of Trauma and Orthopedic Surgery, Addenbrooke’s Hospital, University of Cambridge, Cambridge, UK

**Keywords:** Artificial Intelligence, Small Language Models (SLMs), Clinical Documentation, Knowledge Distillation, Sustainability in AI, AI Agents, Computer science, Scientific data, Software, Health care, Health care economics

## Abstract

The increasing demand for multilingual capabilities in healthcare technology highlights the critical need for AI solutions capable of handling underrepresented languages, such as Arabic, in clinical documentation. Arabic’s unique linguistic complexities—morphological richness, syntactic variations, and diglossia—present significant challenges for foundational large language models (LLMs), especially in domain-specific tasks like medical summarization. This study introduces AraSum, a domain-specific AI agent built using a novel knowledge distillation framework that transforms large multilingual LLMs into lightweight, task-optimized small language models (SLMs). Leveraging a synthetic dataset of Arabic medical dialogues, AraSum demonstrates superior performance over JAIS-30B, a foundational Arabic LLM, across key evaluation metrics, including BLEU and ROUGE scores. AraSum also outperforms JAIS in Arabic-speaking evaluator assessments of accuracy, comprehensiveness, and clinical utility while maintaining comparable linguistic performance as measured by a modified PDQI-9 inventory. Beyond accuracy, AraSum achieves these results with significantly lower computational and environmental costs, demonstrating the feasibility of deploying resource-efficient AI models in low-resource settings for domain-specific tasks. This work underscores the potential of SLM-based agentic architectures for advancing multilingual healthcare, encouraging sustainable artificial intelligence, and fostering equity in access to care.

**Supplementary Information:**

The online version contains supplementary material available at 10.1038/s41598-025-10451-x.

## Introduction

The increasing demand for multilingual capabilities in healthcare technology highlights a crucial need for AI models capable of effectively processing diverse languages, especially in clinical documentation and decision-making. Medical documentation in a patient’s native language can significantly improve understanding, communication, and overall patient care outcomes, particularly in regions where healthcare professionals and patients may not share a common language, or where nuanced medical terminology requires precise understanding. In this regard, Arabic presents unique challenges for language models due to its rich morphological structure, complex syntax, and diglossia^1^, which is the coexistence of formal Arabic and regional dialects. Effective scribing and clinical workflow models that operate seamlessly in Arabic can facilitate accurate documentation, reduce medical errors, and bridge communication gaps that are crucial for patient care.

Arabic poses distinct challenges for large language models (LLMs) that are often trained on clinical information from well-represented Western or Eastern languages, like English or Chinese^2^. Unlike these languages, Arabic is highly inflected, and small changes in root morphology can drastically alter word meanings^3^. In clinical contexts, where precision is paramount, capturing these subtleties can mean the difference between an accurate diagnosis and potential misinterpretation. The research underscores that while natural language processing (NLP) for Arabic has advanced, especially with the introduction of Arabic-specific models like AraBERT^4^ and more recently Inception AI’s foundational model JAIS^5^, the field still lacks models that perform well in specialized medical domains.

Existing Arabic models frequently face challenges in accurately interpreting clinical terminology, where meanings can shift significantly based on context—an issue compounded by the limited availability of comprehensive medical conversation data for validation^6^. Effective clinical workflows and documentation demand precise, consistent, and interpretable summaries. For Arabic-speaking patients and providers, this necessitates a model specifically designed to address linguistic complexities, with a focus on medical terminology and the ability to contextualize and summarize clinical data effectively in Arabic. Furthermore, foundational LLMs are both resource-intensive and often show inconsistent performance on domain-specific tasks^7^, creating significant gaps in the application of AI to medicine, particularly in clinical summarization for non-English languages.

Our team has recently proposed and implemented a new solution to address these holes in clinical AI adoption – instead of relying on bulky foundational models, we harness a novel knowledge distillation framework to create and arrange agents housing small language models (SLMs) in a collaborative architecture. This approach has been shown in prior literature to be more capable on domain-specific tasks, including in metrics of accuracy and efficiency, although conflicting perspectives have been presented in a fine-tuned models’ proclivity to biases compared to foundational models^7–10^. However, to our knowledge, there has been no prior investigation into applying this approach to the medical domain, much less in specialized tasks such as medical summarization or medical Arabic.

Our investigation found that our SLM-based model, AraSum, is potentially more accurate and suited to clinician usage when handling domain-specific medical summarization tasks in Arabic compared to JAIS, the foundational Arabic LLM. Through this study, we show that our SLM-based agentic approach demonstrates superior aspects for conducting domain-specific tasks, such as medical summarization, compared to foundational LLMs. This also has profound ramifications for access-to-care and technological equality in medical artificial intelligence across languages and cultures.

## Methods

### Data source

To overcome the lack of accessible and vouched medical conversation datasets exclusively in Arabic, we adopted a synthetic data generation approach commonly seen in many studies, including an investigation by Al-Mutairi et al., which has shown that synthetic data created by LLMs can suffice as a data source for training and validation of AI models^6,11,12^.

A total of 4,000 Arabic medical conversation transcripts and corresponding ground-truth medical summaries were generated using GPT-4o. The primary criteria for generating each simple clinical vignette featured in the interactive transcripts are summarized in Supplementary Table S1, including medical conditions, patient demographics, symptom characteristics, clinical details, family history, and environmental and social factors. By incorporating these expanded variables, the synthetic dataset samples real-world clinical diversity, enabling the model to learn from a range of scenarios and produce accurate, contextually relevant summaries.

A random subset of the synthetic dataset (~ 20%) was translated into English and then checked for quality assurance by a medical student experienced in both patient management skillsets and clinical research. By doing so, we generated confidence in the accuracy of the dataset and introduced accountable variability in the dataset, allowing robust learning, mimicry of human learning, and resistance to spurious correlations^13,14^.

This study did not involve human participants or identifiable personal data and was therefore not subject to ethical considerations in accordance with 45 CFR 46.102. The dataset used was fully synthetic, generated by an AI model (GPT-4o) without any use of real patient information or records, consistent with legislation and other peer-reviewed studies. According to U.S. regulations, research using no human subjects or identifiable health information does not require IRB approval or informed consent in accordance with 45 CFR 164. Likewise, under EU law, the synthetic data did not constitute personal data per GDPR Recital 26, Article 4, and the EU AI Act’s Article 10 and thus fell outside the scope of data protection and consent requirements. No protected health information (PHI) or real human subjects as defined by HIPAA was used in this work, either to train, fine-tune, or validate any models. In line with regulatory guidance, our use of in silico synthetic medical data posed no privacy risk and warranted no ethics board oversight.

### Development of AraSum SLM agent through knowledge distillation framework

AraSum was created by leveraging a knowledge distillation framework (Supplementary Figure S2) to transform large, multilingual language models into a compact student model optimized for the nuanced task of summarizing patient information in Arabic. This approach retains the performance characteristics of the teacher while reducing computational complexity, making it suitable for deployment in resource-constrained environments^15–22^. After standard pre-processing for whitespace and null entries, a tokenizer capable of handling Arabic texts was utilized to enable accurate semantic and grammatical processing by the model^23^.

Our study employed a multi-teacher distillation approach to enhance Arabic medical text summarization by leveraging two complementary multilingual Transformer models. Teacher Model A, facebook/mbart-large-50-many-to-many-mmt, is a multilingual encoder-decoder Transformer with 12 encoder and 12 decoder layers, pre-trained on a 50-language corpus including Arabic, and chosen for its strong multilingual summarization performance. Teacher Model B, google/mt5-large, is a sequence-to-sequence Transformer with approximately 1.2 billion parameters, pre-trained on the multilingual T5 corpus, effectively handling morphologically rich languages like Arabic. During training, logits from both teachers were independently generated on synthetic Arabic medical transcripts and combined via weighted averaging based on validation performance, creating a unified teacher signal.

The student model, AraSum, specifically targets Arabic medical summarization tasks with a Transformer architecture comprising 12 encoder and 8 decoder layers. AraSum was partially initialized using down-projected weights from teacher models to retain essential lexical and contextual knowledge, thereby accelerating convergence and improving overall performance^24^. Additionally, a specialized SentencePiece tokenizer enriched with medical vocabulary and Arabic diacritics was developed, enhancing AraSum’s linguistic precision.

Our training approach leveraged a multi-teacher distillation framework employing both Kullback-Leibler (KL) divergence and cross-entropy loss functions to guide AraSum in mimicking the ensemble behavior of the teacher models. AraSum was trained using synthetic Arabic medical transcripts, with tokens batched at a global size of 64 per update, achieved by processing mini batches of 16 tokens across approximately 4 gradient accumulation steps. Training was conducted with a peak learning rate of 1 × 10⁻⁴, incorporating a warm-up phase of 500 steps. The model typically underwent 8–10 epochs of training, with early stopping triggered if the validation loss plateaued over two consecutive epochs. Regularization methods included a dropout rate of 0.1 in both attention and feed-forward sublayers, complemented by a weight decay of 0.01 to enhance generalization. The training process was executed on multiple NVIDIA A100 GPUs^25^ in parallel, leveraging PyTorch’s Distributed Data Parallel (DDP) framework. Each training cycle typically lasted between 5 and 8 h, depending on dataset complexity and the total number of epochs.

For validation, performance was systematically evaluated using metrics such as ROUGE and BLEU scores. Checkpoints were monitored continuously, with the best-performing model selected based on the highest F1 score from a held-out validation set. Our current implementation centers around a singular integrated AraSum agent: a unified system internally orchestrates various tasks associated with medical note generation and summarization, rather than relying on multiple discrete agent entities.

### Evaluation of model accuracy using known quantitative metrics

The synthetic dataset was split at a 90:10 ratio for training and validation. AraSum and JAIS-30B were both tasked using zero-shot prompting to create summaries of each clinical conversation transcript. To ensure clarity and grammatical accuracy in clinical summaries, the model and tokenizer were configured to preserve and generate Arabic diacritics (Taksheel/Harakat) as needed. The following quantitative metrics were calculated from the generated summaries on the validation set.

The “Evaluate” library^26^ was used to calculate all aforementioned scores for each sample. Clinical content recall was defined as the proportion of relevant clinical information from the ground truth summary accurately captured in the AI-generated summaries. Salient clinical items were extracted from each conversation into an inventory. The recall was then calculated by dividing the number of correctly included items from the inventory by the total number of relevant items. Clinical content precision was defined as the proportion of information that was both accurate and relevant when compared to the ground truth. Precision was calculated by dividing the number of correctly included items in the summary by the total number of items, including any additional or incorrect items. This metric reflects the accuracy and relevance of the content without introducing extraneous or inaccurate details. The F1 score is used as a balanced metric to combine both clinical content precision and recall, providing a single measure of the AI-generated summaries’ performance^27^. It represents the harmonic mean of precision and recall, ensuring that both the accuracy of relevant information captured (precision) and the completeness of that information (recall) are considered. Finally, ROUGE scores^28^, BLEU^29^, and BERTScore F1^30^ were also calculated based on their original methodologies.

### Measuring clinical utility with Arabic-fluent evaluators and modified performance inventories

Three random transcripts of synthetic patient-physician conversations were extracted from the dataset, along with their respective ground truths, AraSum-, and JAIS-generated summaries (Supplementary Figure S3). Eight evaluators who speak Arabic were consulted for this study, including four healthcare professionals comprised of two medical students, one of which who was a former Arabic medical interpreter, a radiologist, and a clinical professor. Consent to collect evaluator’s feedback data and publish was obtained. Each were provided the transcripts and the JAIS- and AraSum-generated summaries, blinded to the source. Summary quality was evaluated using a modified version of the Physician Documentation Quality Instrument 9 (PDQI-9). The original PDQI-9 employs a 5-point Likert scale across nine attributes to assess note quality. This was modified by Tierney et al. into a ten-item inventory to better fit the metrics relevant to ambient AI documentation and is widely used to evaluate AI-generated clinical notes^31,32^. Three additional language-specific attributes, syntactic proficiency, domain-specific linguistic precision, and cultural competence were added to the inventory for the Arabic evaluators to assess the model’s ability to generate language as native Arabic speakers do. The attributes evaluated in this modified PDQI-9 are detailed in Table 1.

### Statistical analysis

For comparing ROUGE and BLEU scores, normality was tested in the distribution of all scored metrics using the Shapiro-Wilk test, then a paired non-parametric comparison between the two models was conducted for each metric using the Wilcoxon signed rank test in GraphPad Prism version 10.4.1 for macOS (GraphPad Software, Boston, MA, www.graphpad.com). Cohen’s d value for paired data was calculated by dividing the mean of the paired differences by the standard deviation of all paired differences. The rank-biserial correlation was computed as the difference between the proportion of pairs where AraSum outperformed JAIS and the proportion of pairs where JAIS outperformed AraSum, divided by the total number of paired comparisons. Both analyses were conducted in Python (ver. 3.9). For comparing attribute scores from the modified PDQI-9 inventory, normality was assumed through the central limit theorem and law of large numbers, and results were compared through a paired Student’s t-test in Microsoft Excel. All graphs were generated using GraphPad Prism or the Seaborn and Matplotlib libraries in Python.

## Results

### AraSum outperforms JAIS in AI-centric measures of clinical summarization performance

**Fig. 1:**
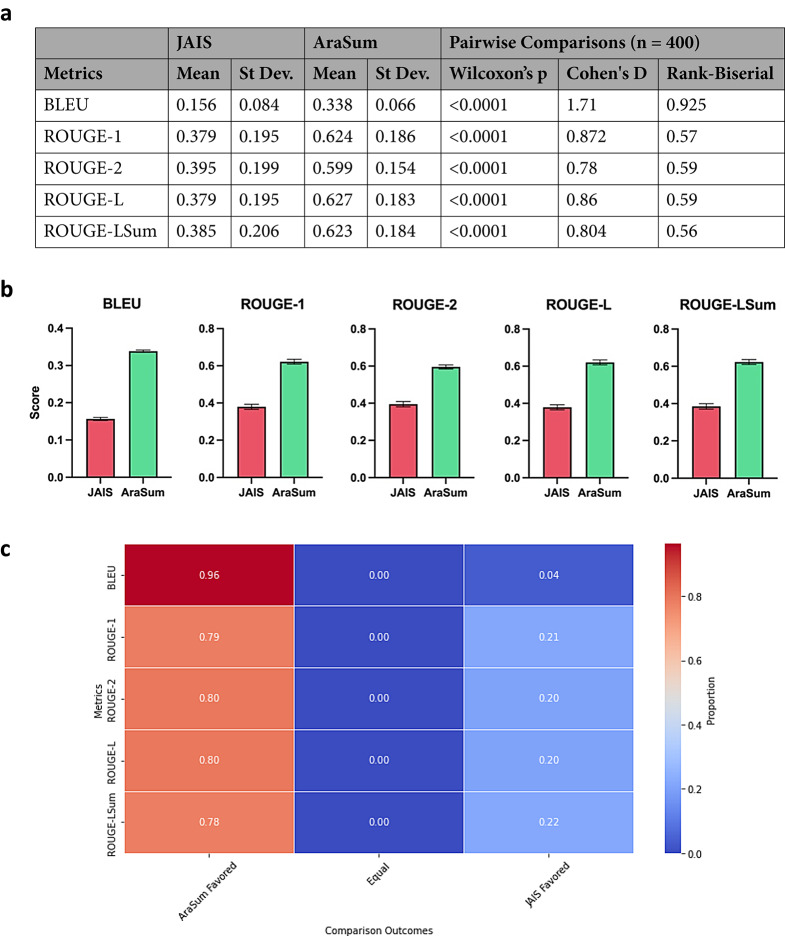
AraSum outperforms JAIS in lexical, semantic, and phrase-level metrics of summary quality. (a) BLEU and ROUGE scores for JAIS and AraSum and pairwise comparison statistics. (b) Bar graphs for BLEU and ROUGE scores with reported standard error bars. (c) Pairwise comparison analysis heatmap shows proportions of scores that favor either AraSum or JAIS or are equal.

The metrics included in the analysis are common industry-standard quantities for measuring AI performance and demonstrate key differences between AraSum and JAIS’ capabilities. Individual BLEU and ROUGE scores across the validation set (*n* = 400) were averaged, then plotted in a distribution for further analysis (Fig. 1A, B).

AraSum achieved a mean BLEU score of 0.338 (95% CI: [0.332, 0.344]), significantly higher than JAIS’s mean of 0.156 (95% CI: [0.145, 0.164]), with a p-value of < 0.0001. The Cohen’s d value of 1.71 and a rank-biserial correlation of 0.925 indicate a strong effect size favoring AraSum. For reference, a Cohen’s d value magnitude above 0.8 and rank-biserial magnitude above 0.5 is considered a large effect^33^. The BLEU score, which measures the precision of n-gram overlaps between the generated and ground-truth summaries, highlights AraSum’s superior ability to replicate ground-truth summaries with higher fidelity.

Further analysis using ROUGE scores revealed a more granular perspective on the distinct performance capabilities between AraSum and JAIS. When comparing ROUGE-1 to assess for lexical accuracy and matching key terms, AraSum’s mean was 0.624 (95% CI: [0.606, 0.642]), compared to JAIS’s 0.379 (95% CI: [0.360, 0.398]), with a p-value of < 0.0001, a Cohen’s d of 0.872, and a rank-biserial correlation of 0.57. The capture of short context windows and meaningful word pairings was assessed by comparing the two models’ ROUGE-2 scores, with AraSum scoring 0.599 (95% CI: [0.584, 0.614]), significantly surpassing JAIS’s 0.395 (95% CI: [0.375, 0.415]) with a Cohen’s d of 0.78 and a rank-biserial correlation of 0.59.

Similarly, AraSum excelled in ROUGE-L (mean 0.627, 95% CI: [0.609, 0.645]) compared to JAIS (mean 0.379, 95% CI: [0.360, 0.398]), indicating higher structural preservations in AraSum’s summaries. Furthermore, ROUGE-LSum, a metric specifically created for summarization tasks, also indicated that AraSum (mean 0.623, 95% CI: [0.605, 0.641]) significantly outperformed JAIS (mean 0.385, 95% CI: [0.365, 0.405]) in overall summary quality.

A pairwise comparison analysis (Fig. 1C) shows that AraSum outperformed JAIS across all statistics in most (> 78%) of the pairings of AI-generated summaries. This was further mirrored in content-dependent metrics of AI performance (Supplementary Table S4), such as AraSum’s BERTScore F1 was 0.807, compared to JAIS’s 0.724, indicating a higher semantic similarity to ground-truth summaries. AraSum also demonstrated superior recall (0.549 vs. 0.160) and precision (0.557 vs. 0.364), with an F1 score of 0.552 compared to JAIS’s 0.220.

Statistical significance has been established in all BLEU and ROUGE scores (*p* < 0.0001), alongside significantly large effect size through Cohen’s d value and the rank-biserial, overall indicating that the differences measured in the two performances can be attributed to differences in the capabilities between AraSum and JAIS.

### Arabic-fluent clinical evaluators rank AraSum higher in clinical utility attributes

The clinical utility of AraSum versus JAIS was assessed through our modified PDQI-9 inventory tailored specifically for both summary quality and linguistic performance. Across the eight Arabic-fluent evaluators, Table 2 delineates the average scores (*n* = 24) for each attribute.

Both models performed exceptionally well in their ability to synthesize language in the summaries as given by their similarly high scores in language-specific attributes. However, AraSum achieved a significantly higher (*p* = 0.007) mean score of 4.21 (95% CI: [3.81, 4.60]) for “Accurate” compared to JAIS’ 3.63 (95% CI: [3.11, 4.14)). Similarly, AraSum was rated as more “Thorough,” with a mean score of 4.58 (95% CI: [4.31, 4.86]) versus 3.17 (95% CI: [2.67, 3.66]) for JAIS (*p* < 0.001). It can be reasoned that AraSum generates more precise and complete summaries of patient data, enhancing its reliability and utility for clinical decision-making.

AraSum was rated as significantly more “Useful” (*p* = 0.001) with a mean score of 4.38 (95% CI: [4.05, 4.70]) compared to JAIS’ mean score of 3.33 (95% CI: [2.84, 3.83]). Additionally, AraSum was deemed more comprehensible (*p* = 0.002), scoring 4.42 (95% CI: [4.20, 4.63]) compared to JAIS’ 3.83 (95% CI: [3.45, 4.22]). These findings suggest that AraSum provides summaries that are both more relevant and easier to understand for end-users.

When further comparing the ability to generate medical summaries with accurate, consistent reasoning, AraSum outperformed JAIS in terms of “Synthesizing” information effectively (*p* = 0.008), with a mean score of 4.17 (95% CI: [3.80, 4.53]) versus 3.50 (95% CI: [3.07, 3.93]). It was also rated higher for “Internally Consistent” (AraSum = 4.25, 95% CI [3.99, 4.51]; JAIS = 3.83, 95% CI [3.47, 4.20]; *p* = 0.005), indicating that AraSum produces outputs that are more cohesive and logically structured.

An interesting result presented by the evaluations was that AraSum was evaluated higher (mean = 4.21, 95% CI [3.84, 4.58]) “Free from Bias” attribute compared to JAIS (mean = 3.79, 95% CI [3.22, 4.36]) with statistical significance (*p* = 0.047). While prior literature that worked with fine-tuned models have reported emerging biases^8^, these results suggest that AraSum’s knowledge distillation framework may mitigate the risk of biases compared to foundational models.

While AraSum scored higher in several other attributes, these differences were not statistically significant. This can be interpreted as having comparable performances in these categories. This has an important implication that the AraSum SLM has comparable linguistic performances to JAIS while maintaining its domain-specific task expertise. It is also notable that AraSum’s performance in providing bias- and hallucination-free summaries was comparable to that of the foundational model, further bolstering confidence in its capabilities and potential for adoption.

## Discussion

To our best knowledge, our study is the first investigation to utilize a knowledge distillation framework to create fine-tuned SLMs specialized for medical domain-specific tasks in Arabic. The findings suggest that AraSum, born successfully from this development workflow, can be superior in accuracy, clinical utility, comprehensiveness, usability, consistency, and knowledgeability for task-specific purposes with no sacrifices to linguistic quality compared to foundational LLMs.

Amongst its superior performance ratings by Arabic speaking evaluators, the most intriguing and controversial is AraSum’s higher scores in reduced biases. There are conflicting perspectives that argue for and against the potential of bias in this framework – some argue that it is possible for knowledge distillation to be harnessed to reduce bias due to the learning of “softer” probability distributions^34,35^, while others have reported amplified biases^36^. Nevertheless, especially within these clinical contexts, both the AI-centric measures of summary quality and speaker-evaluated attributes seemed to favor the utility of the specialized lightweight model.

The potential ramifications of these results are contextually important in the developing world of adoptable artificial intelligence. Building foundational models such as GPT-3, with 175 billion parameters, would cost $4.6 million per training run at the lower limit, and modern models such as GPT-4, with over 1.76 trillion parameters (~ 7 terabytes in total GPU memory) and trained on 13 trillion tokens, cost OpenAI well over $100 million for development^37,38^. JAIS’ most complex model has 30 billion parameters and was trained on 1.63 trillion tokens of Arabic and English text. By similar cost calculations, this was also a multi-million dollar and three-week investment for training alone^5,39,40^. These foundational models are groundbreaking developments for the pursuit of knowledgeable, general-purpose AI, but the staggering costs make them inaccessible and economically infeasible for implementation in each domain-specific task. This is especially exacerbated for in-house or cloud-based implementations in larger medical institutions, where necessary infrastructure, maintenance workforce, and usage training must be carefully delineated and heavily financed before being adopted by clinicians^41^.

The growing problems surrounding the cost of development are further exacerbated by the environmental footprint associated with the heavy computational burden. Training GPT-3 harnessed multiple NVIDIA V100 GPUs working in parallel to manage all 175 billion parameters. A study by Anthony et al. estimated that a single training run for GPT-3 harnessing the most powerful V100 GPUs, the V100S PCIe model, at a Microsoft data center translates to approximately 2.8 × 10^4^ GPU-days. The energy conversion is approximately 1.9 × 10^5^ kWh of energy or 8.5 × 10^4^ kg of CO_2_ – equivalent to driving a car over 700,000 km^42^. Other estimates place the total impact as high as around 5.5 × 10^5^ kg^43^. Furthermore, taking into account both the operational usage of ChatGPT, which is estimated to be around 1.0 × 10^7^ queries per day, as well as estimating that GPT is retrained once per month, the combined impact of ChatGPT amounts to around 2.2 g CO_2_ per query^44^.

The carbon footprint surrounding the training of JAIS is not publicly available. However, it is known that JAIS was trained on the Condor Galaxy (CG-1) supercomputer designed for high-efficiency training^5^. Although these supercomputers were designed for high computing efficiency that leads to lower emissions than conventional setups, the computational burden of training 30 billion parameters would lead to significant energy costs and carbon footprint.

In contrast to these foundational models, harnessing existing open-source LLMs to utilize in training SLMs-based agentic models has only a fraction of the development costs or carbon footprint. AraSum’s training took place over several days on NVIDIA A100 GPUs, with additional weeks dedicated to further fine-tuning and validation. Development costs were also only a small fraction compared to foundational models. Across ten training runs, each amounting to 5–8 h, in a USA-based data center gives a power estimate of 1.6 kW x 50–80 h. = 80–128 kWh. This translates to around 40–64 kg CO_2_ in total emissions for the creation of AraSum. Even with this low cost and impact development, AraSum demonstrated superior domain-specific task performance with comparable linguistic capabilities as JAIS-30B, giving valuable proof-of-concept to the cost-efficiency of this approach in adopting artificial intelligence to solve niche problems.

The implications of this study are not only relevant to Arabic-speaking regions but also have broader implications for the development of multilingual AI models in healthcare. The methodologies behind AraSum’s creation serve as a blueprint for creating language-specific models that address the unique linguistic and cultural challenges of underrepresented languages. Such advancements are critical in bridging communication gaps in diverse healthcare settings, enabling better patient outcomes and reducing disparities in care delivery. Prior literature has established language barriers as key obstacles to healthcare access and equality in Arabic-represented venues such as the United Arab Emirates and Saudi Arabia^45,46^. Thus, this study opens doors to the possibilities of cost-efficient artificial intelligence for resolving these critical healthcare gaps.

Given the potential of AraSum and similar lightweight models in resource-constrained or underserved healthcare environments, discussing strategies for scalable deployment across various infrastructure types is essential. For instance, adopting an open approach^47^ leverages common edge devices such as hospital workstation access units (WAUs) or mobile devices. However, these devices typically face constraints in computational power, memory, and energy efficiency compared to cloud-based solutions. Nevertheless, Nissen et al. demonstrates that deploying inference tasks on mobile devices is viable, with memory constraints identified as a more significant limitation than processing capability^48^. Consequently, lightweight models derived through knowledge distillation, such as AraSum, are highly attractive for edge deployments. Future research should explore techniques like 8-bit or 4-bit quantization and pruning to further minimize memory usage, alongside comparative analyses of quantization-aware training (QAT) versus GPTQ methods to optimize inference speed, accuracy, and memory efficiency.

Conversely, in larger-scale clinical environments such as major academic medical centers, cloud-based deployments utilizing closed-approach microservices offer distinct advantages^47^. AraSum’s inherently low computational and development costs further contribute to reduced overall deployment expenses. Its existing architecture already supports distributed inference, facilitating horizontal scaling through additional AraSum instances to accommodate increased demand with minimal overhead. These lightweight models, developed on similar frameworks as AraSum, can be deployed on leading hyperscalers as serverless endpoints within broader cloud-based applications, which works synergistically with AraSum’s lightweight nature to further decrease carbon footprints of model training and usage^41,49^.

However, cloud-based deployments inherently carry risks related to data exposure and breaches beyond hospital-controlled environments, especially to external stakeholders or cybersecurity attacks^2^. As an alternative, hybrid models such as “cloud-bursting,” combining cloud service providers (CSPs) and on-premise infrastructure, offer reduced data exposure and latency, aligning better with healthcare data privacy requirements^41^.

Regarding data privacy, federated learning (FL) emerges as an effective approach to addressing privacy concerns related to centralized patient data management in healthcare research^50^. FL enables institutions to collaboratively train machine learning models without directly sharing raw data, thus safeguarding patient confidentiality and complying with regulatory frameworks such as HIPAA and GDPR. In practice, local models are trained using institution-specific datasets, with only model updates or aggregated metrics shared externally. Federated learning has successfully been implemented across multiple healthcare domains and demonstrates substantial promise for fostering collaborative research while upholding stringent data privacy standards^51^.

While AraSum demonstrates significant improvements over JAIS, this study relies heavily on synthetic data due to the scarcity of real-world clinical datasets in Arabic. While synthetic data has proven effective for evaluation, future research should incorporate real-world clinical conversation datasets to validate these findings further. This may involve collecting a dataset of clinical conversations in primarily Arabic-speaking practices from large institutions or collection of physician volunteers, then utilizing a transcription agent to develop conversation transcripts, paired with standardized summaries generated real-world Arabic-speaking clinicians. This dataset may help to fine-tune AraSum to capture nuances of genuine patient-provider interactions.

Additionally, expanding evaluations to include a wider variety of clinical scenarios and integrating user feedback from a larger diversity of Arabic-speaking healthcare professionals would provide a more comprehensive assessment of the model’s practical utility. Investigations into several other AI-based Arabic solutions and comparisons to Arabic-speaking clinicians in summarization performance are also warranted for an even more robust confirmation of AraSum and its development workflow. Further exploration is also needed into the integration of AraSum into clinical workflows, particularly in developing seamless interfaces for real-time documentation and decision support. Finally, expanding the model’s capabilities to encompass other dialects and regional variations of Arabic could enhance its applicability across different Arabic-speaking populations.

Nevertheless, this study provides valuable insights into the potential of SLMs in ameliorating the healthcare landscape: in clinical workflow, for the sustainability of adoptable artificial intelligence, and across global cultures. Sporo AraSum is one example of unlocking the capabilities presented in the frontiers of artificial intelligence. These tailored models are not only accurate and useful but also possess the ability to transform multilingual healthcare by bridging language barriers, decreasing costs and environmental impact, and optimizing clinical workflows.

**Table 1:** Items of the modified PDQI-9 for AI-generated summary evaluation.

PDQI-9 Attribute	Explanation
Accurate	The note does not present incorrect information.
Thorough	The information presented is comprehensive and lacks omissions. It contains all information that is relevant to the patient.
Useful	The information presented is relevant and provides valuable information for patient management.
Organized	The note is formatted in a way that is coherent and easy to comprehend. It helps the reader to understand the patient’s story and the management of their clinical case.
Comprehensible	The note is straightforward, with no unclear sections.
Succinct	The note does not contain redundant information and presents relevant information in a concise, direct manner.
Synthesized	The note demonstrates the AI’s comprehension of the patient’s condition and its capability to formulate a care plan.
Internally Consistent	The facts presented within the note are consistent with each other and do not contradict the patient’s story, each other, or medical knowledge.
Free from Bias	The note is unbiased and includes only information that can be verified by the transcript, without being influenced by the patient’s characteristics or the nature of the visit.
Free from Hallucinations	The information in the note aligns with the content of the transcript, without any factual inaccuracies or AI-generated hallucinations.
Syntactic Proficiency	The note correctly uses grammar, relevant vocabulary, and sentence structure to construct accurate sentences as a native speaker would.
Domain-Specific Linguistic Precision	The note correctly uses medical vocabulary and domain-specific phrasing that best fits the presented clinical scenario. The note utilizes language that native clinicians would use to write their note.
Cultural Competence	The note’s language accurately reflects and respects the cultural, linguistic, and social nuances in Arabic. This includes understanding of culturally specific health beliefs or practices, OR the way that language is utilized surrounding medicine itself. (For example, “palliative care” is not an explicit concept defined in Arabic, or many serious illnesses such as cancers are instead referred to as “the illness.”)

**Table 2:** Comparison of model performances across AI-generated summaries.

PDQI-9 Attribute	AraSum	JAIS	p-value (*n* = 24)
Accurate	4.21	3.63	**0.007**
Thorough	4.58	3.17	**< 0.001**
Useful	4.38	3.33	**0.001**
Organized	3.92	3.33	0.055
Comprehensible	4.42	3.83	**0.002**
Succinct	4.21	3.96	0.314
Synthesized	4.17	3.50	**0.008**
Internally Consistent	4.25	3.83	**0.005**
Free from Bias	4.21	3.79	**0.047**
Free from Hallucinations	4.13	3.75	0.095
Syntactic Proficiency	3.96	4.00	0.788
Domain-Specific Linguistic Precision	4.00	3.83	0.162
Cultural Competence*	4.05	3.76	0.110

*One evaluator opted out from evaluating “Cultural Competence” due to their stance on diverse cultures and beliefs surrounding medical Arabic across different countries and regions. For this attribute, 21 evaluations across the remaining seven evaluators and three samples were analyzed.

## Electronic supplementary material

Below is the link to the electronic supplementary material.

Supplementary Material 1

## Data Availability

The original synthetic dataset and performance data can be shared upon reasonable request and execution of a Data Usage Agreement. The AraSum model can be accessed via Hugging Face upon reasonable request, but model weights and parameters are strictly proprietary. Please reach out to corresponding author, CL, for any inquiries.
