# Supplementary material for: Democratizing cost-effective, agentic artificial intelligence to multilingual medical summarization through knowledge distillation
Source: Sci Rep. 2025 Jul 29;15:27619. doi: 10.1038/s41598-025-10451-x (PMC12307869; doi:10.1038/s41598-025-10451-x)
Supplement: Supplementary file 1 — Supplementary Material 1 [file 41598_2025_10451_MOESM1_ESM.docx]

**Democratizing cost-effective, agentic artificial intelligence to multilingual medical summarization through knowledge distillation**

**Authors**

Chanseo Lee,*^1,2^ Sonu Kumar MTech,^1^ Kimon A. Vogt MS,^1^ Muhammad Munshi,^2^ Panindhra Tallapudi,^1^ Antonia Vogt,^3^ Hamzeh Awad PhD,^4^ Wasim S. Khan PhD FRCS^5^

1. Sporo Health, Boston, MA, United States

2. Department of Surgery, Yale School of Medicine, New Haven, CT, United States

3. Girton College, University of Cambridge, Cambridge, United Kingdom

4. Faculty of Allied Medical Sciences. Middle East University, Amman, Jordan.

5. Department of Trauma and Orthopedic Surgery, Addenbrooke’s Hospital, University of Cambridge, Cambridge, United Kingdom

*Corresponding author

Corresponding Author Email: chanseo.lee@yale.edu

**SUPPLEMENTARY INFORMATION**

| Variable | Explanation |
| --- | --- |
| Chief complaints | Acute conditions such as صداع )headache), حمى )fever), ألم في البطن )abdominal pain), سعال )cough), ضيق في التنفس )shortness of breath)… or chronic/complex disease states such as أمراض القلب )heart diseases), أمراض القلب )kidney diseases), أمراض الرئة المزمنة )chronic respiratory diseases), اضطرابات نفسية )psychiatric disorders). |
| Age group | طفل )child), مراهق )adolescent), شاب )young adult), متوسط العمر )middle-aged), مسن )elderly). |
| Gender identity | ذكر )male), أنثى )female), غير محدد )non-binary). |
| Severity | خفيف )mild), متوسط )moderate), شديد )severe). |
| Medications | Wide range of medications employed for each clinical vignette. |
| Prior Medical History | تاريخ طبي سابق مثل العمليات الجراحية )surgical history), الأمراض المزمنة )chronic illnesses), الحساسية للأدوية )drug allergies)… |
| Lifestyle Factors | Lifestyle Factors: التدخين )smoking), تناول الكحول )alcohol consumption), النشاط البدني )physical activity), نظام عذائي )diet)… |
| Family History | Inclusion of familial history details for applicable clinical vignettes. |
| Occupational Hazards | تعرض لمواد كيميائية )chemical exposure), إجهاد عمل )work-related stress). |
| Socioeconomic Status | مستوى التعليم )education level), الدخل )income level). |

**Supplementary Table S1.**

Variables for generation of synthetic clinical conversation and summary dataset.


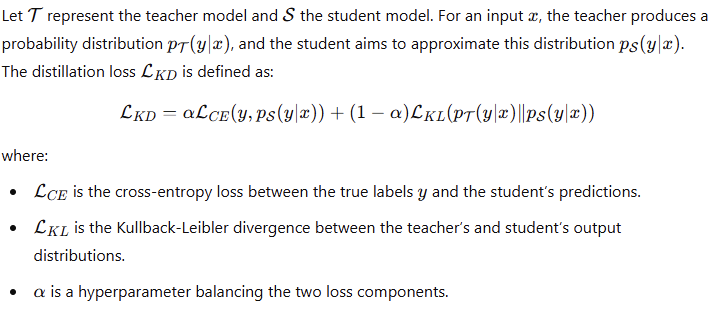


**Supplementary Figure S2.**

Mathematical formulation of the knowledge distillation utilized for the creation of AraSum.


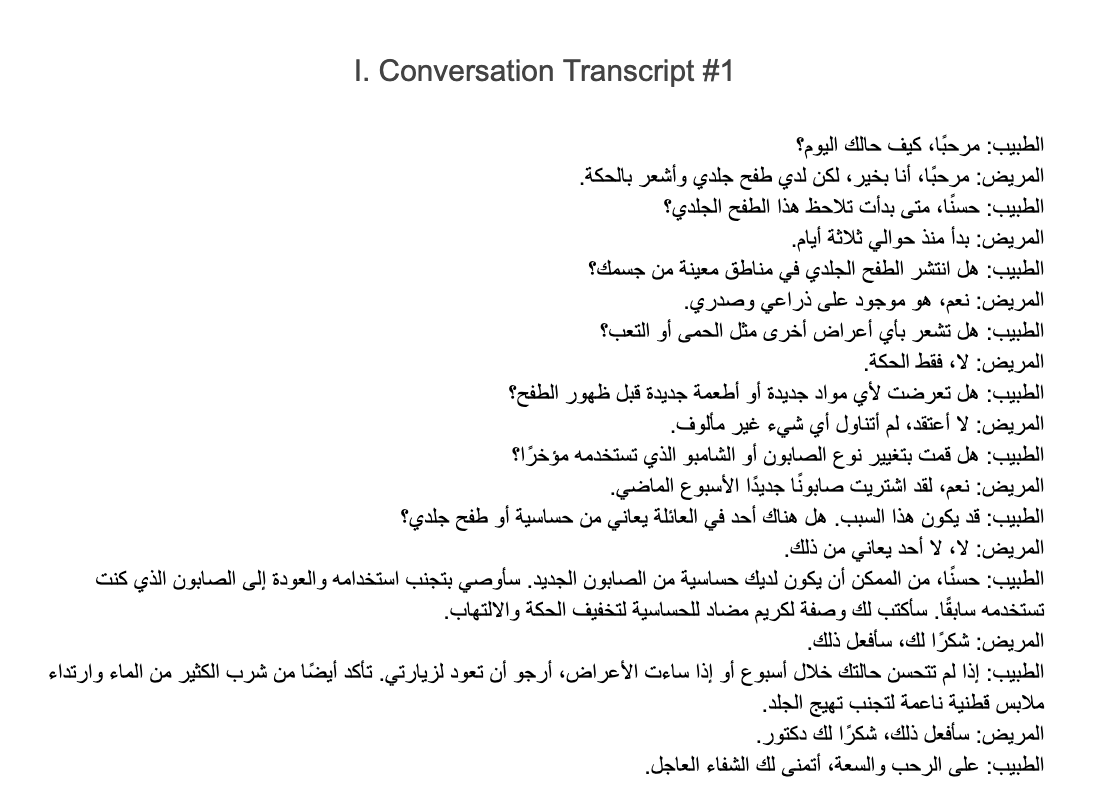


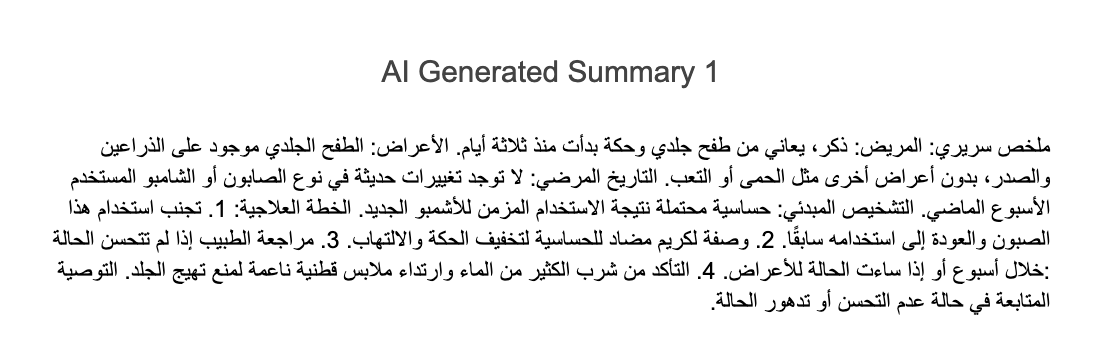

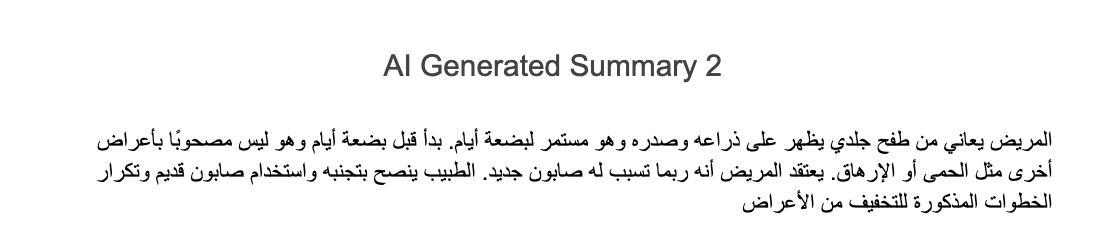


**Supplementary Figure S3.**

Example Conversation Transcript and AI-Generated Summaries.

Summary #1 is by AraSum, Summary #2 is by JAIS.

| Metric | JAIS | AraSum |
| --- | --- | --- |
| Precision | 0.364 | 0.557 |
| Recall | 0.160 | 0.549 |
| F1 Score | 0.220 | 0.552 |
| BERTScore F1 | 0.724 | 0.807 |

**Supplementary Table S4.**

Additional content-based metrics of AI summary quality
